# Gender inequities in women’s access to maternal health care utilisation in Zambia: a qualitative analysis

**DOI:** 10.1186/s12884-023-06078-3

**Published:** 2023-10-26

**Authors:** Audrey M. Kalindi, Brian Houle, Bruce M. Smyth, Vesper H. Chisumpa

**Affiliations:** 1 School of Demography, The Australian National University, Acton, ACT Australia; 2 MRC/Wits Rural Public Health and Health Transitions Research Unit (Agincourt), Faculty of Health Sciences, School of Public Health, University of the Witwatersrand, Johannesburg, South Africa; 3 Institute of Behavioral Science, University of Colorado Boulder, Boulder, CO USA; 4 Centre for Social Research and Methods, The Australian National University, Acton, ACT Australia; 5 Department of Population Studies, School of Humanities and Social Sciences, The University of Zambia, Lusaka, Zambia

**Keywords:** Maternal health care utilisation, Gender inequities, Power relations, Zambia

## Abstract

**Background:**

The role of gender inequities in women’s ability to access maternal health care has mainly been analysed from either women’s or men’s perspective only. In this article, we explore the role of gender inequities in maternal health care utilisation from both men’s and women’s perspectives.

**Methods:**

Thirty-six interviews were conducted with reproductive age women (*n* = 24), and men whose wives/partners gave birth within the last three years prior to our study in Zambia (*n* = 12). Our study sought to improve understanding of the normative environment in which women and men make decisions on maternal health care utilisation in Zambia.

**Results:**

We found that men and women had different expectations regarding their gender roles in maternal health care utilisation, which created inequities reinforced by societal norms and traditions. Men make most household decisions including those related to reproductive health and they often have the major say in access to maternal health services despite not having holistic maternal health information which creates challenges in maternal health care utilisation.

**Conclusion:**

The study highlights the need for maternal health care utilisation decisions to be made by both men and women and that men should be fully involved in maternal health care from pregnancy until after child birth. Further, there is urgent need for concerted and sustained efforts to change traditional norms that reinforce these inequities and affect maternal health care utilisation if Zambia is to meet Sustainable Development Goal-3.1.

**Supplementary Information:**

The online version contains supplementary material available at 10.1186/s12884-023-06078-3.

## Background

Maternal and child health are global health priorities and continue to be major population health challenges in low and middle-income countries (LMIC). Globally, an estimated 800 women die every day due to causes related to pregnancy and childbirth [1]. A defining characteristic of the many maternal and child health studies is the “assumption of women’s primacy in maternal health care utilisation” [2]. As a result, most of the literature on maternal and child health is based on women’s perceptions, attitudes and experiences of health care utilisation and health outcomes [3–5]. However, maternal and child health is a multidimensional issue that requires a collective effort by everyone – including men. In sub-Saharan Africa (SSA), men are regarded as heads of households and they ultimately make most household decisions including health care [6]. In recent years, there has been a growing body of maternal and child health research targeting men’s involvement [7, 8].

Most LMIC have strong social structures that rigorously define men’s and women’s roles, which are frequently encoded in religious, tribal and social traditions [9]. Gender encompasses the socially constructed norms, behaviours, and roles linked to being male or female, as well as the dynamics between individuals within a given society. These norms can exhibit significant variation across societies and evolve with time, reflecting the intricate interplay of culture and societal shifts [10]. Similarly, gender discrimination denotes the unequal treatment or prejudice against individuals based on their gender, often leading to inequities in opportunities, access to resources, and decision-making power. Gender discrimination can act as a barrier in maternal health care utilisation as evident in the male-centred nature of many reproductive health supplies and services [11]. Women’s unique needs and perspectives are often overlooked, for instance, contraceptives, have historically been designed with a male-centric focus, limiting women’s choices and control over their reproductive health [12]. Addressing gender disparities in maternal healthcare access and outcomes is crucial for achieving equitable and improved maternal health for women [10].

Gender roles bring with them the potential for gender discrimination between men and women. Gender discrimination has been shown as a barrier to high-quality maternal and newly born health care as men make most household decisions including reproductive health such as the type of contraceptive to use even if it may have side effects on the woman [5, 13]. There is also an inherent gender discrimination in health service delivery. A number of health care providers, for instance, prioritise men’s decision-making authority over women’s reproductive autonomy [13]. Women, especially younger and less educated, fail to exercise their household freedom and decision-making autonomy sufficiently to obtain quality maternal health services [14].

Among the sub-Saharan African (SSA) countries, Zambia holds a significant place for research due to the reinforcement of gender inequities by cultural and traditional norms, which have become deeply ingrained and widely accepted within the community [4–8]. These entrenched norms have had a profound influence on the perpetuation of gender disparities in the country. The significance of examining gender dynamics in Zambia lies in the need to comprehend the intricate interplay between these deeply ingrained cultural norms and the resulting gender disparities. This research is instrumental in shedding light on the complex factors shaping the Zambian context. These two districts chosen have had a lot of donor interventions [15, 16] and conducting gender inequities in maternal health care utilisation brings out aspects that typical socioeconomic determinants may fail to capture as maternal and child mortality is still high in these provinces [17]. Existing gender inequity maternal health studies in Zambia have looked at maternal health care use from women’s perspective only [18, 19]. By contrast, others have looked at male involvement in maternal health from a male perspective [7, 20]. Only a few studies have looked at both perspectives in tandem but without considering how gender relations and household dynamics work to affect maternal health care utilisation [21]. To date, few studies have applied an in-depth qualitative lens to household dynamics that play a role in maternal health care utilisation. Rarely have they explicitly examined how such power differences shape maternal health care utilisation [22–24]. Yet this lens has clear implications for understanding maternal health care utilisation at household and community levels that have strong patriarchal systems and gender inequities which is predominant in SSA [25–28]. This study therefore seeks to improve understanding of the extent to which men’s perspectives of gender-related barriers are similar to those of women in Zambia and if they differ, in what ways. It further seeks to explore how the interplay of both women and men’s perspectives at household and community levels can influence maternal health service utilisation among women in Zambia.

The present study aims to address these gaps by exploring men and women’s perspectives on how gender dynamics (decision-making power, access to resources, division of labour and social norms) play a role to influence maternal health care access and utilisation at the individual, institutional and community levels in Zambia. It further examines the extent to which each partner’s position and role in a relationship determines the relative decisions and use of maternal health services. A unique feature of the present study is the use of in-depth interviews and the Gender Analysis Framework (GAF) [29, 30].

## Methods

### Study setting

The study took place in two districts, Mansa and Kalomo in Luapula and Southern Provinces of Zambia respectively. These districts were chosen due to their location in provinces that have different socioeconomic factors and the highest under five and child mortality rates (48 and 110 deaths per 1000 live births, respectively in Luapula). Southern province is one of the two provinces that had the highest neonatal mortality rates (NMR) in Zambia in 2018 (33 deaths per 1000 live births). From 2013 to 2018, Zambia’s NMR increased from 24 to 27 deaths per 1000 live births [31]. Health facilities included in the study were assessed according to the number of deliveries (health facility and home) recorded in 2020 (the year prior to the study), as both health facility and home deliveries were included (See Supplementary material 2).

### Study population

Two groups of participants were purposively selected to gain comprehensive insight into maternal healthcare services. The groups include 24 women who delivered at the health facility and at home, as well as 12 men whose partners delivered at the health facility and at home. This selection aimed to ensure a wide representation, encompassing women from both urban and rural areas, as well as those residing near healthcare facilities and those in more remote locations. These comprised women in reproductive ages (15–49 years) who had their previous birth as a home or healthy facility delivery and men whose wives or partners delivered from health facilities or home. For ethical reasons and practicality in data collection, the number of women was double that of men as we wanted to get more insights on the birthing experience from both women who delivered from home and at the health facilities (See Supplementary material 2). Further, women who had a miscarriage or foetal death within the year of study were excluded from the study.

### Design

The study used an interpretive case study design. Individual in-depth interviews focused on demand, access and utilisation of maternal health care services, and socioeconomic effects of maternal morbidity and mortality. Gender dynamics are operationalised as relationships and interactions between and among women and men based on gender (being men or women). The study investigates how men and women’s relationships and interactions affect maternal health care utilisation. We hypothesised that access and utilisation of maternal health care services is influenced by the social norms within the community, and these determine who makes rules and decisions and how the division of labour occurs in households and communities. Mothers’ and fathers’ experiences and perspectives regarding maternal health services were captured to gain contextual understanding of their perceptions and experiences of maternal health care utilisation.

### Sampling

Districts were divided into health facilities with better maternal and child health indicators and those with poor indicators such as health facility type and equipment based on routine administrative data from Health Management Information System (HMIS) [32]. Distance was also factored in based on how far the health facility is from the population catchment area by either walking or using a vehicle and whether it was in a rural or urban area. The health facilities that allowed people to walk to access them were regarded as near and those that needed people to use a car were considered as far, and further divided by being in an urban or rural area and whether they are basic emergency obstetric and new born care (BEmONC) or comprehensive obstetric and new born care (CEmONC) facilities. This was done to gain wider perspectives from the maternal and child health stakeholders. Study participants were recruited with the assistance of Maternal and Child Health Coordinators as well as health facility staff and Safe Motherhood Action Groups (SMAGs) based on pre-set selection criteria that included having lived experiences of or having a partner who has lived experiences of maternal health services, having had a delivery or a partner who had a delivery either at home or at the health facility in the past three years (See Supplementary material 1 and 2).

### Data collection

In-depth participant interviews were used to collect data between August and September, 2021 at the peak of the Covid-19 pandemic (see Supplementary material 1). Three Research Assistants (RAs) with experience in qualitative research collected the data. English interview guides with open-ended questions and probes were used. The RAs translated them into local languages, Bemba (Mansa District) and Tonga (Kalomo District), for participants who did not understand English.

### Data processing and analysis

All interviews were audio-taped with consent from study participants, and transcribed verbatim by the principal investigator and the RAs. The principal investigator listened to each recording and read through the transcripts repeatedly to gain an overall sense of the data and formulate the study themes.

**Fig. 1:**
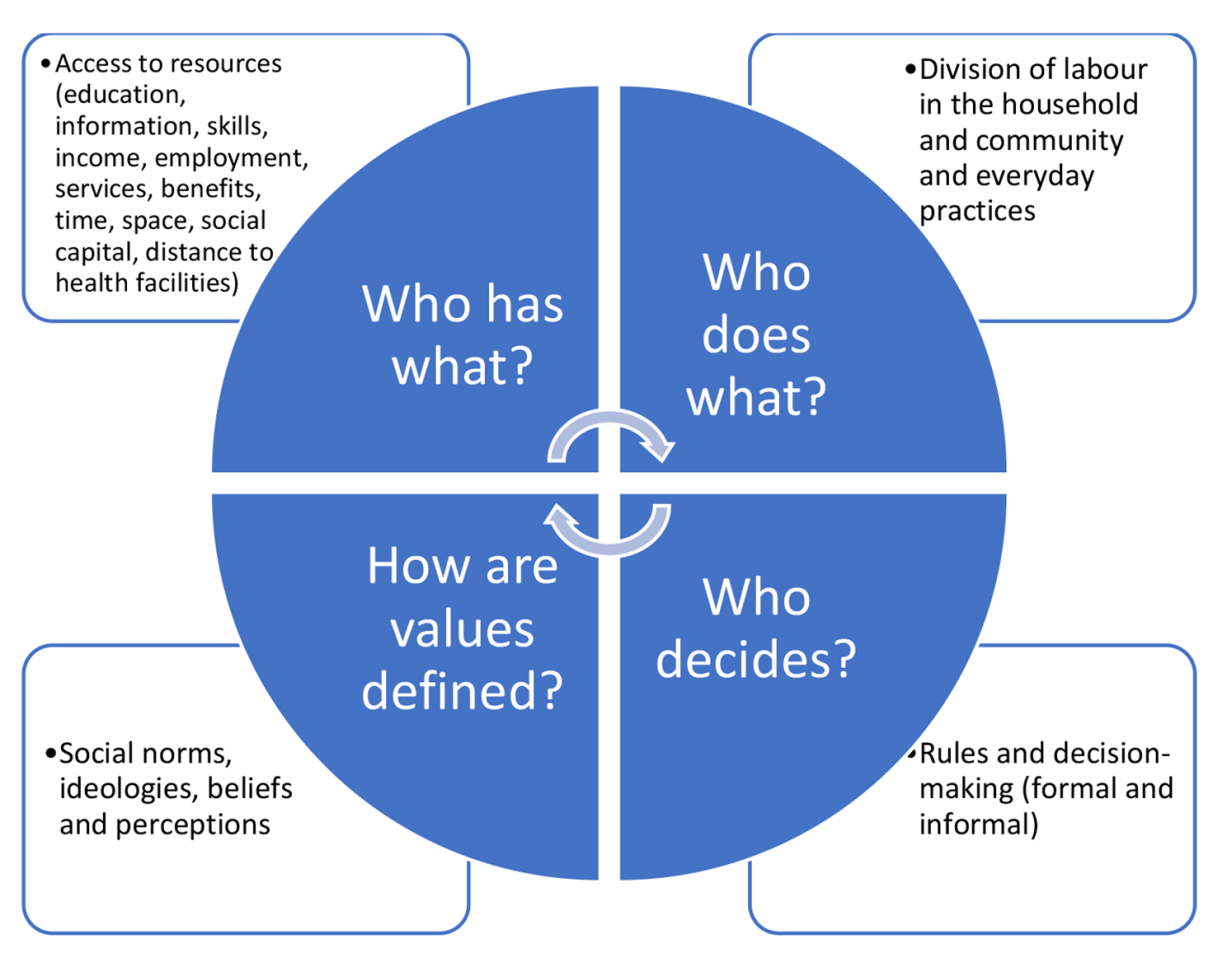
Adapted Gender analysis Framework. Adapted from “A Guide to Gender analysis Framework” [33]

Transcripts were exported to NVivo software for analysis. A thematic content transcript analysis approach in tandem with the GAF [30, 33] was used to identify and code salient themes that emerged from the data. The study utilised a gender analysis framework to explore disparities in maternal healthcare utilisation (See Fig. 1). This framework is used to improve understanding of gender-related issues and dynamics such as gender roles and identities, power and decision-making, access to resources and intersectionality within a particular context. It seeks to uncover gender inequities, and opportunities for promoting gender equality and women’s empowerment, especially in the context of programme implementation and evaluation. Grounding decision-making in empirical evidence and data, gender analysis enhanced accountability, advocacy efforts, and advanced the pursuit of health equity in line with SDGs [29] for a patriarchal country such as Zambia [34]. The constant comparative method was used to compare findings systematically and to ensure the validity of the study results [35]. Guided by the conceptual framework and interview guides, both deductive and inductive coding approaches were applied and each code was further analysed and disaggregated into categories and sub-themes. The framework was adapted to mothers’ and fathers’ experiences and perspectives regarding how maternal health care utilisation is determined by access, social norms, division of labour and decision-making power.

## Results

### Background characteristics

A total of 36 interviews were conducted comprising 12 women who delivered from home; 12 who delivered from a healthy facility; 6 men whose partners delivered from home and 6 whose partners delivered from a health facility. Supplementary material 2 shows that twice the number of women participated compared with men (*n* = 24 vs. 12). The mean age of mothers was 29 years for Kalomo and 26 years for Mansa while the age ranged from 15 to 49 years and the mean age of males for Kalomo was 36 and 38 for Mansa. Most mothers had not received any formal education although a few completed primary and secondary school (4–12 grades). This was also the case for husbands, although some had some tertiary education. Farming was the dominant occupation; other participants were shopkeepers and arts men. Most men were older than their wives.

### Inequities in women’s continuum of maternal health care utilisation

Through the gender analysis framework lens, the analysis of inequities in maternal healthcare utilisation in Zambia revealed four key thematic areas: (a) barriers related to access to essential resources, (b) the significance of rules and decision-making dynamics, (c) the influence of deeply ingrained social norms and community beliefs, and (d) the critical role of the division of labour and household head’s provision, all of which emphasise the pronounced gender disparities inherent in this context.

### Access to resources

Access to resources implies that women should be able to have health care as and when they need it, and it should be appropriate for everyone. Both men and women reported that a number of women failed to access maternal health care due to their low socioeconomic status as most of them were unable to meet the costs associated with childbirth. As a result, women that did not deliver from the health facilities mostly did not prepare supplies that health facilities require for them to deliver from there. For instance, one of the women who delivered at home stated:

*“I did not have what I needed to take to the clinic for delivery. Some women may not afford to buy anything for the baby. In my case, I decided to stay home because I may be ashamed if I go to the clinic”* (Interview 2).

Men shared a similar view. For instance, one husband whose wife gave birth at home said:

*“Poverty is a big problem for us during antenatal, delivery and post-natal care because all of the stages require money for transport, buying items in readiness for delivery and also buying healthy food for our wives is a challenge”* (Interview 3).

Another husband whose wife delivered at a health facility remarked:

*“When we go for antenatal, we are told to prepare for delivery and are asked to come with delivery materials, i.e. baby blanket, dish, gloves, jik (antiseptic), plastic and even the mother needs supplies such as pads. I managed to source most of these items for my wife but it was very expensive as I had to forgo certain household supplies or hold buying things for the other children as we had to prioritise this baby” (Interview 27 ).*

Likewise, a woman who delivered at home reflected:

*“I delivered at home because of fear to go to the health facility as the materials/supplies were not adequate. So, I was afraid that I may be shouted at- at the health facility”* (Interview 5).

Financial autonomy has remained a factor in maternal health care utilisation over time. A number of women in the study reported that finances were a barrier to health care utilisation. A woman who delivered from the health facility reported that:

*“It depends on the partner you have. Some don’t manage to buy what is required and health personnel say if you don’t have the requirements, we’ll send you to the general hospital so that they know what to do. Because some are not married and are drunkards who just got pregnant and are scared to point out the person responsible, so she’ll just stay back in the village and won’t give birth at the hospital”* (Interview 7).

The socioeconomic status of their partners also determined their choice and access of health care utilisation. This was reported by both men and women. If a husband did not keep money for transportation to go and deliver from the health facility, it was difficult for the partner to go on their own unless she was economically empowered. As some women reported, they delivered at home because they did not have supplies to go and deliver at the health facility. According to a husband whose wife delivered at home:

*“We had prepared most of the things needed for my wife to deliver at the health facility but I did not prepare for transport. I was not home when labour started and by the time I reached home, I found that she had already delivered”* (Interview 35).

Participants reported issues of distance to the health facility as a barrier to health care as some of the women could not attend the required number of antenatal visits or deliver from a health facility because their husbands did not provide transportation in time. The women ended up either delivering at home or on their way to the health facility. As one home birth mother remarked:

“*Like here in district X, we should have a nearby health post where we should take our children for under five clinic but we don’t”* (Interview 17).

According to one husband whose wife delivered at the health facility, there is also long waiting times at health facilities:

*“We waited for a long time at the health facility to receive care for my wife. There was only one medical staff and there were a lot of people waiting to be attended to”* (Interview 11).

### Rules and decision making

Women and men were asked how decisions are made in the household including major decisions such as maternal health care utilisation. Depending on the household, decisions were made together, by the women only, or her partner only. Rules refer to the written and unwritten regulations that societies and families live by and eventually use to make household decisions. Participants reported that most women sought their husband’s approval in health care utilisation even though men reported that they make decisions together with their spouses. Some women who did not have a supportive husband made decisions on their own as they did not have anyone to consult. Both men and women indicated that most men have the final say regarding household decisions including health care utilisation. As much as some women may wish to make their own maternal health decisions, there are other layers affecting decision making such as financial resources. Most women, for example, depend on their husbands who have to provide for them during pregnancy as well as prepare for child birth and secure the baby’s needs [36]. As was reported by a man whose wife had a home birth:

*“It is me who makes decisions and I tell her to say, woman, since the pregnancy has matured, go and stay at the health facility”* (Interview 11).

Another man whose wife delivered from the health facility remarked:

*“We need to save some money or have money in advance, so that immediately when something happens, I know how to move to access health care for my wife*” (Interview 12).

A woman who delivered at home reported:

*“Much as we make decisions together, my husband has the final say. I had prepared for my delivery well in advance but when labour started, I tried to contact my husband who was not home but by the time he arrived, I had already given birth”* (Interview 34).

Rules and decisions were also a significant factor if there was a large age difference between the husband and wife. Most women interviewed were five years younger than their husbands on average, and this may also affect health care decisions as the wife is mostly expected to comply with the husband’s choices. One woman who delivered at the health facility remarked:

*“My husband is the head of the house and we follow the decisions he makes as they are in the best interest of the family”* (Interview 14).

### Social norms and community beliefs and perceptions

Communities have shown to have a major influence on women’s birthing choices [37]. This is very common in rural areas where village leaders are highly respected and carry considerable influence on their people. Social norms, beliefs and perceptions play a major role in these kind of societies [36]. Most successful government programmes have had to go through the village headmen and leaders as they have a lot of influence on their subjects and whatever is conveyed to the people through them has a high probability of success. As reported by a man whose wife delivered at home, generally, men are known to be household providers including in the domain of health care, where the man’s role is viewed as that of provider.

*“In our society, it is the duty of a man to be head of the household and to provide for the family and the woman is expected to take care of the home”* (Interview 26).

The study shows that men and women had different expectations in terms of partner support as what men thought was holistic support was not adequate in relation to what women expected from their partners. For many men, they felt financial support was enough for the woman as their family role was that of provider. As one man whose wife had a health facility delivery reported:

*“I support my wife financially and that is the duty of a husband. I made sure she had all the supplies that she needed to deliver from the health facility”* (Interview 16).

Most women in the study reported that the support was not enough as they expected more help from their partners beyond financial support. Some women reported lack of partner support as a reason for failure to seek maternal health care as stated by a woman who delivered at a health facility:

*“When I was pregnant, my husband still expected me to do the normal household work and when I took time to rest and sleep, he said I was lazy. This made me so tired and it was difficult to attend antenatal care”* (Interview 23).

A number of women suffer from various pregnancy related diseases throughout their pregnancy and tend to be so weak such that they need to take rest often but their partners think that they are just lazy. They are expected to continue doing normal household chores and also help out in the fields, some as far as nine months into their pregnancy. As more women who delivered at home reflected:

*“My husband still expected me to work in the fields even when my pregnancy was advanced and it was too hard for me as I was supposed to cook for him too and be a mother to my other children”* (Interview 18).

*“When labour started, my husband had gone to play football and I just started walking to the health facility and he found me on the way to the clinic as labour had started”* (Interview 6).

*“Except for the time we went to register the pregnancy which is mandatory for men, when it’s time to learn on how to take care of the pregnancy, nutrition during pregnancy, malaria prevention and postnatal care, I go alone. We women are the ones who learn about pregnancy and exactly understand how to take care of ourselves during pregnancy as compared to men who hardly accompany us”* (Interview 9).

### Division of labour and household head provision

In addition to social norms, division of labour and family provision by the head of household have shown to influence women’s utilisation of maternal health care services. Even though women identified lack of partner support as contributing to low maternal health care utilisation, most men reported that they supported their partners in totality and both the women and their babies did not lack support from them. The apparent mismatch in expectations tends to compromise the expected support partners give their wives during pregnancy, (ranging from household chores to health care utilisation support like accompanying the wife for antenatal and postnatal visits) because partners do know the ideal support to offer [38].

As reported by a man whose wife delivered from the health facility:

*“I asked my mother to come and help my wife during pregnancy”* (Interview 11).

Another man whose wife had a home birth reflected:

*“When my wife was pregnant, her mother came over to help her with the pregnancy and how to take care of the baby”* (Interview 16).

The study shows that men tend to report that they are totally supportive of their wives during pregnancy and child birth whereas women stated that husbands accompanied them in few instances for antenatal, rarely for postnatal and delivery sometimes but most of the work is done by the women as reflected by one woman who gave birth at home.

*“I delivered at home because there was no one to escort me to the health facility”* (Interview 29).

This lack of support is re-echoed by a woman who delivered at the health facility:

*“I had a maternal complication and was expected to go for medical check-ups more than the regular times required for antenatal in a normal pregnancy. Because of distance and no one to escort me, I did not manage to go for the required number of check-ups the nurse asked me to do”* (Interview 21).

Arranging transport is often regarded as a male responsibility and so if women deliver from home because of not having transportation, then the male partners are regarded as failing their duties. One of the males whose wife delivered from the health facility reported:

*“Some women fail to seek maternal health care services because of their partners. If my partner is pregnant, I need to encourage her to seek these services. As men, sometimes we consume too much alcohol and do a lot of gambling instead of taking care of our families”* (Interview 10).

Providing transportation to the health facility was often perceived as not being enough because whereas men felt as if they were doing a favour, women believed this to be their minimum responsibility. A man whose wife delivered at the health facility reported:

*“I helped my wife a lot, I cleaned the house and took care of the children when she was pregnant”* (Interview 22).

Emotional and physical support came out strongly among women as something they lacked from their partners as reported by a woman who had a home birth:

*“Some of our friends are escorted by their partners and they take very little time as men are prioritised by health care staff since they go out of their way to support their wives despite being busy people” (*Interview 17).

Additionally, men and women are expected to do certain chores in a household. As much as some men are helpful, even when a woman is pregnant, most men still expect their wives to do strenuous house chores. This makes it hard for women to seek maternal health care as they are usually very busy with things like farm work, clearing the land and weeding. A woman who delivered at the health facility reported:

*“My husband says he cannot eat food that is prepared by another person and I have to prepare all the three meals from breakfast to supper as his wife even when I am too tired”* (Interview 24).

Some of these women who do these chores have adverse maternal complications and may end up having miscarriages and/or other morbidities due to the strenuous work that they do. This is echoed by women who delivered from home:

*“Even if we do all the house chores, we are made to do other heavy tasks like working in the fields and collecting firewood for cooking”* (Interview 33).

*“A woman has lot of responsibilities at home such as cleaning cooking, fetching water and so on, to a point where sometimes I forget that I need to go for antenatal care”* (Interview 15).

## Discussion

In this paper, we explored men and women’s perspectives of how gender dynamics interact at the household level to impact maternal health care utilisation and examined the extent to which each partner’s position and role in the relationship determines the relative decisions and use of maternal health services in Zambia. Overall, we found that gender dynamics play a role at multiple levels to bring about inequities that limit maternal health care access and utilisation in Zambia. Despite having well defined societal gender roles, men and women have different expectations which affect how they negotiate to make health-care decisions and utilisation in the household. Societal norms, cultures and traditions reinforce gendered traditions that discourage maternal health care utilisation by limiting women’s autonomy [38, 39]. Women feel that they must live according to societal expectations even if it may sometimes compromise their health and that of their infants [39–41].

By bridging scholarship on population health and research on the gendered inequities in maternal health care utilisation, our study makes three key contributions. Firstly, gender inequities raise several mutually reinforcing factors that affect maternal health care utilisation. These inequities are inherent at household, community and institutional levels and are supported by traditional norms and beliefs that affect maternal health care utilisation [42]. Maternal health has been regarded as an issue for women, yet women do not make key decisions in the household [43]. On the other hand, the men who mostly make these decisions are typically unaware of holistic maternal health information. This means their decisions may not always be in the best interest of the health of women and their infants [43].

Secondly, society has defined men and women’s roles and responsibilities which households have tried to adhere to though not fully. These roles and responsibilities come with challenges such as financial for males who may fail to provide for their homes and some women who may fail to do household chores because of sickness experienced during their pregnancy. Many women in LMIC have low literacy levels and lack equal job opportunities as their male counterparts [28, 44]. This has resulted in high dependency on men by women as the only means of survival [45]. Most women, especially those in rural areas, depend on their husbands or partners for financial support [46, 47]. This applies to health care utilisation as well as other costs related to seeking health care [46]. This means that men are also implicated in the process as they are more financially independent than women [48]. These expectations create gaps that have affected decision making regarding health care utilisation and have led to low maternal health care utilisation [48]. Men and women, however, wish they were supported by their partners in ways not defined by society but may positively influence maternal health care utilisation. Some women have ended up developing long-term illnesses and others have died due to heavy household chores given to them by their partners or families, in the event they live with other family members [49].

Third, our study extends prior scholarship on masculinity and femininity by examining how gendered societal expectations affect maternal health care utilisation. For many men, the low maternal health care utilisation among women was not seen as partly their failure to fulfil their traditional provider role but blamed on harsh economic conditions. Conversely, women discuss low health care utilisation more in terms of their partners not being supportive or failing to provide for them when it is not of their own making but due to other challenges. In each case, gender inequities show how these interweaving factors affect maternal health care utilisation in addition to structural factors in Zambia.

Analysing maternal health utilisation through a gender perspective shows obscure environments within which maternal health-related decisions are made and how these decisions can affect maternal health care utilisation. Despite indications of women making decisions together with their husbands, the study reveals that men had more decision-making power including financial resources in the household. With maternal health care services depending significantly on finances and husband decision making, men to a larger extent determine when and what type of maternal health care women and children receive [48]. This affects women’s health care utilisation, and dependency on their male partners which might delay health care seeking, as women mostly depend on the husband’s financial status and how responsive they are towards timely maternal health care seeking [20, 50, 51]. The decision-making factor extends to women who may be financially independent as they do not make ultimate household decisions [4, 52, 53]. The unfortunate part is that some of these women might have maternal complications and cannot afford to wait for the delayed health care access [54].

To address gender inequity, our results suggest a community approach may yield more positive results. The GAF shows that society has a tremendous role in determining norms which to a large extent determine decisions made in the household as well as accessibility and utilisation of maternal health care. These approaches have worked in most Asian and SSA countries like Zambia where the community has a large influence on household and individual norms and decisions including maternal health care utilisation [39, 47, 55]. There are certain gender roles expected by societies which if men and women did not follow, they would be deemed not worthy of that society [56]. This influences their decision-making processes including health care. Women’s autonomy, education and having individual sources of income have been shown to empower women financially and help them make better health decisions [57].

Three study limitations warrant mention. Data were drawn from a small non-probability purposive sample and no claim is made that key findings generalise to other parts of the country or region. In addition, the GAF used in the study down plays other factors that may affect maternal health care utilisation (e.g. its potential oversimplification of gender, cultural variability, neglect of men and boys, and a primary focus on the individual level rather than broader structural factors) which have been shown to influence maternal health care utilisation across many regions in the world [58, 59]. The data nonetheless provide powerful insights into both men’s and women’s perspectives on maternal health care utilisation inequities.

## Conclusion

This study highlights the role of gender and how it interacts with socioeconomic factors in producing and sustaining limitations to women’s access of quality health care. Most approaches to women’s health, including those done in SSA, have incorrectly assumed equalisation of power and access to resources for women and men [60, 61]. It foregrounded women’s perceptions and experiences to understand multiple forms of oppression that intersect to create health care utilisation. They also reveal important gender dynamics and how these create barriers to women’s access to and utilisation of maternal healthcare in Zambia. The study has shown that women’s limited decision-making power and low socioeconomic status exacerbated gender norms in restricting their use of health care.

Men’s role in maternal health care utilisation was found to be key as they are identified as final decision-makers in women’s access to care, largely determined by social norms that predict gender roles and unequal division of labour within households. Socioeconomic status also determined the kind of maternal health care received as poorer households had more challenges in health care accessibility and utilisation. Multiple layers such as strenuous household and farm work coupled with financial restraints and low literacy levels restricted women’s access to skilled health care. As the country endeavours to achieve SDG 3, successful interventions to improve women’s health care utilisation and health outcomes should be holistic and target multiple factors that promote inequities in women’s financial independence, literacy levels, gender roles and decision making power at both household and community levels for both men and women [38, 42]. Interventions that challenge gendered norms and attitudes can help to break down barriers to maternal healthcare access and utilisation especially in rural Zambia.

This study has not only focused on the differences between men and women, but went further to explore how gender as a power relationship drives inequality and limits access to health systems among women. The findings illustrate how gender inequities affect access and utilisation of maternal health care services in Zambia. Future research should expand this study to see how it unfolds at country level and should explicitly analyse how gender affects maternal health care utilisation for women and men with different levels of education and employment.

More broadly, the study suggests the need to involve boys and men in education and awareness regarding the importance of maternal health care and emotional support for women during pregnancy and childbirth. This is especially important in Zambia, where men traditionally play significant roles in decision-making processes. Involving boys is crucial because cultural norms and traditions are often established from a young age and tend to persist overtime.

### Electronic supplementary material

Below is the link to the electronic supplementary material.

Additional file 1: Supplementary Materials 1, 2 and 3.

## Data Availability

The materials for data collection are available on request from the corresponding author. The datasets for this study are not publicly available to preserve participants’ confidentiality, however, de-identified data can be provided for the purposes of reproducibility and data veracity if requested from the corresponding author.

## References

[1] UNICEF. UNICEF DATA. 2023 [cited 2023 Jun 15]. Maternal Mortality. Available from: https://data.unicef.org/topic/child-survival/under-five-mortality/.

[2] Mondal D, Karmakar S, Banerjee A (2020). Women’s autonomy and utilization of maternal healthcare in India: evidence from a recent national survey. PLoS ONE.

[3] Yaya S, Bishwajit G, Shah V (2016). Wealth, education and urban–rural inequality and maternal healthcare service usage in Malawi. BMJ Glob Health.

[4] Namasivayam A, Osuorah DC, Syed R, Antai D (2012). The role of gender inequities in women’s access to reproductive health care: a population-level study of Namibia, Kenya, Nepal, and India. Int J Womens Health.

[5] Kriel Y, Milford C, Cordero J, Suleman F, Beksinska M, Steyn P (2019). Male partner influence on family planning and contraceptive use: perspectives from community members and healthcare providers in KwaZulu-Natal, South Africa. Reprod Health.

[6] Ganle JK, Obeng B, Segbefia AY, Mwinyuri V, Yeboah JY, Baatiema L. How intra-familial decision-making affects women’s access to, and use of maternal healthcare services in Ghana: a qualitative study. BMC Pregnancy Childbirth. 2015;15. 10.1186/s12884-015-0590-4 PMC4537557 26276165

[7] Yaya S, Okonofua F, Ntoimo L, Udenigwe O, Bishwajit G (2019). Men’s perception of barriers to women’s use and access of skilled pregnancy care in rural Nigeria: a qualitative study. Reprod Health.

[8] Greenspan J, Chebet J, Mpembeni R, Mosha I, Mpunga M, Winch P et al. Men’s roles in care seeking for maternal and newborn health: a qualitative study applying the three delays model to male involvement in Morogoro Region, Tanzania. BMC Pregnancy Childbirth. 2019;19. 10.1186/s12884-019-2439-8 PMC6693212 31409278

[9] Omer S, Zakar R, Zakar MZ, Fischer F (2021). The influence of social and cultural practices on maternal mortality: a qualitative study from South Punjab, Pakistan. Reprod Health.

[10] WHO. Gender and health [Internet]. 2023 [cited 2023 Sep 5]. Available from: https://www.who.int/health-topics/gender.

[11] Roudsari RL, sharifi F, Goudarzi F (2023). Barriers to the participation of men in reproductive health care: a systematic review and meta-synthesis. BMC Public Health.

[12] Campo-Engelstein L (2012). Contraceptive justice: why we need a male pill. AMA J Ethics.

[13] Oduenyi C, Banerjee J, Adetiloye O, Rawlins B, Okoli U, Orji B (2021). Gender discrimination as a barrier to high-quality maternal and newborn health care in Nigeria: findings from a cross-sectional quality of care assessment. BMC Health Serv Res.

[14] Kassahun A, Zewdie A (2022). Decision-making autonomy in maternal health service use and associated factors among women in Mettu District, Southwest Ethiopia: a community-based cross-sectional study. BMJ Open.

[15] Central Statistical Office, University of Zambia Department of Population Studies, Centers for Disease Control and Prevention, Centers for Disease Control and Prevention, United States Agency for International Development, ICF International., Zambia Saving Mothers, Giving Life Maternal Mortality Endline Census in Selected Districts [Internet]. 2017 [cited 2022 Oct 1]. Available from: https://www.medbox.org/pdf/5e148832db60a2044c2d5abb.

[16] MoH. 2022–2026 Zambia National Health Strategic Plan [Internet]. 2022 [cited 2023 May 29]. Available from: https://www.moh.gov.zm/?p=3138.

[17] Zambia Statistics Agency, Ministry of Health, University Teaching Hospital Virology Laboratory, ICF International. Zambia - Demographic and Health Survey 2018 [Internet]. 2020 [cited 2021 Nov 30]. Available from: https://microdata.worldbank.org/index.php/catalog/3597.

[18] Mweemba C, Mapulanga M, Jacobs C, Katowa-Mukwato P, Maimbolwa M (2021). Access barriers to maternal healthcare services in selected hard-to-reach areas of Zambia: a mixed methods design. Pan Afr Med J.

[19] Sserwanja Q, Mukunya D, Nabachenje P, Kemigisa A, Kiondo P, Wandabwa JN (2022). Continuum of care for maternal health in Uganda: a national cross-sectional study. PLoS ONE.

[20] Greenspan JA, Chebet JJ, Mpembeni R, Mosha I, Mpunga M, Winch PJ (2019). Men’s roles in care seeking for maternal and newborn health: a qualitative study applying the three delays model to male involvement in Morogoro Region, Tanzania. BMC Pregnancy Childbirth.

[21] Sialubanje C, Massar K, Horstkotte L, Hamer DH, Ruiter RAC. Increasing utilisation of skilled facility-based maternal healthcare services in rural Zambia: the role of safe motherhood action groups. Reprod Health. 2017;14. 10.1186/s12978-017-0342-1 PMC5504812 28693621

[22] Zhang L, Xue C, Wang Y, Zhang L, Liang Y. Family characteristics and the use of maternal health services: a population-based survey in Eastern China. Asia Pac Fam Med. 2016;15. 10.1186/s12930-016-0030-2 PMC5081876 27795694

[23] Mullany BC, Becker S, Hindin MJ (2007). The impact of including husbands in antenatal health education services on maternal health practices in urban Nepal: results from a randomized controlled trial. Health Educ Res.

[24] Yamin AE, Bazile J, Knight L, Molla M, Maistrellis E, Leaning J (2015). Tracing shadows: how gendered power relations shape the impacts of maternal death on living children in sub Saharan Africa. Soc Sci Med.

[25] Jousse L. Discrimination and gender inequalities in Africa: what about equality between women and men? [Internet]. Institut du Genre en Géopolitique. 2021 [cited 2023 Sep 5]. Available from: https://igg-geo.org/?p=3863〈=en.

[26] Chirowa F, Atwood S, Van der Putten M (2013). Gender inequality, health expenditure and maternal mortality in sub-saharan Africa: a secondary data analysis. Afr J Prim Health Care Fam Med.

[27] Sikweyiya Y, Addo-Lartey AA, Alangea DO, Dako-Gyeke P, Chirwa ED, Coker-Appiah D (2020). Patriarchy and gender-inequitable attitudes as drivers of intimate partner Violence against women in the central region of Ghana. BMC Public Health.

[28] Jayachandran S (2015). The roots of gender inequality in developing countries. Annu Rev Econ.

[29] Morgan R, George A, Ssali S, Hawkins K, Molyneux S, Theobald S (2016). How to do (or not to do)… gender analysis in health systems research. Health Policy Plan.

[30] Warren H (2007). Using gender-analysis frameworks: theoretical and practical reflections. Gend Dev.

[31] ZSA, Ministry of Health Zambia, ICF. Zambia Demographic and Health Survey 2018. Lusaka, Zambia and Rockville, Maryland, USA. ; 2019. 2019.

[32] Biemba G, Chiluba B, Yeboah-Antwi K, Silavwe V, Lunze K, Mwale RK (2017). A Mobile-Based Community Health Management Information System for Community Health Workers and their supervisors in 2 districts of Zambia. Glob Health Sci Pract.

[33] March C, Smyth I, Mukhopadhyay M. A guide to gender-analysis Frameworks. Oxfam GB; 1999.

[34] Honkavuo L (2021). Women’s experiences of cultural and traditional health beliefs about pregnancy and Childbirth in Zambia: an ethnographic study. Health Care Women Int.

[35] Colak FZ, Van Praag L, Nicaise I (2019). A qualitative study of how exclusion processes shape friendship development among turkish-belgian university students. Int J Intercult Relat.

[36] Ahmed F, Oni FA, Hossen SS (2021). Does gender inequality matter for access to and utilization of maternal healthcare services in Bangladesh?. PLoS ONE.

[37] Cerrato J, Cifre E. Gender Inequality in Household Chores and Work-Family Conflict. Front Psychol [Internet]. 2018 [cited 2022 Sep 11];9. Available from: https://www.frontiersin.org/articles/10.3389/fpsyg.2018.01330. 10.3389/fpsyg.2018.01330 PMC6086200 30123153

[38] Doyle K, Kazimbaya S, Levtov R, Banerjee J, Betron M, Sethi R (2021). The relationship between inequitable gender norms and provider attitudes and quality of care in maternal health services in Rwanda: a mixed methods study. BMC Pregnancy Childbirth.

[39] Buser JM, Moyer CA, Boyd CJ, Zulu D, Ngoma-Hazemba A, Mtenje JT (2020). Cultural beliefs and health-seeking practices: rural zambians’ views on maternal-newborn care. Midwifery.

[40] Jimoh AO, Adaji SE, Adelaiye H, Olorukooba AA, Bawa U, Ibrahim HI (2018). A cross-sectional study of traditional practices affecting maternal and newborn health in rural Nigeria. Pan Afr Med J.

[41] Fleming PJ, Agnew-Brune C (2015). Current trends in the study of gender norms and Health behaviors. Curr Opin Psychol.

[42] Morgan R, Tetui M, Muhumuza Kananura R, Ekirapa-Kiracho E, George AS (2017). Gender dynamics affecting maternal health and health care access and use in Uganda. Health Policy Plan.

[43] Gebeyehu NA, Gelaw KA, Lake EA, Adela GA, Tegegne KD, Shewangashaw NE (2022). Women decision-making autonomy on maternal health service and associated factors in low- and middle-income countries: systematic review and meta-analysis. Womens Health.

[44] Ma J, Grogan-Kaylor AC, Lee SJ, Ward KP, Pace GT (2022). Gender inequality in low- and Middle-Income countries: associations with parental physical abuse and moderation by child gender. Int J Environ Res Public Health.

[45] Krishnan S, Dunbar MS, Minnis AM, Medlin CA, Gerdts CE, Padian NS (2008). Poverty, gender inequities, and women’s risk of human immunodeficiency Virus/AIDS. Ann N Y Acad Sci.

[46] Habib SS, Jamal WZ, Zaidi SMA, Siddiqui JUR, Khan HM, Creswell J (2021). Barriers to Access of Healthcare Services for Rural women—applying gender Lens on TB in a Rural District of Sindh, Pakistan. Int J Environ Res Public Health.

[47] Evans A. History lessons for gender Equality from the Zambian Copperbelt, 1900–1990. Gend Place Cult. 2014;22.

[48] Alemayehu M, Meskele M (2017). Health care decision making autonomy of women from rural districts of Southern Ethiopia: a community based cross-sectional study. Int J Womens Health.

[49] CDC. Physical Job Demands– Reproductive Health [Internet]. 2022. Available from: https://www.cdc.gov/niosh/topics/repro/physicaldemands.html.

[50] Aborigo RA, Reidpath DD, Oduro AR, Allotey P (2018). Male involvement in maternal health: perspectives of opinion leaders. BMC Pregnancy Childbirth.

[51] Comrie-Thomson L, Gopal P, Eddy K, Baguiya A, Gerlach N, Sauvé C (2021). How do women, men, and health providers perceive interventions to influence men’s engagement in maternal and newborn health? A qualitative evidence synthesis. Soc Sci Med.

[52] Acharya DR, Bell JS, Simkhada P, van Teijlingen ER, Regmi PR (2010). Women’s autonomy in household decision-making: a demographic study in Nepal. Reprod Health.

[53] UNICEF, Voices of Y. 2019 [cited 2023 Oct 10]. Culture and my survival as a young woman. Available from: https://www.voicesofyouth.org/blog/culture-and-my-survival-young-woman.

[54] Tesha J, Fabian A, Mkuwa S, Misungwi G, Ngalesoni F (2023). The role of gender inequities in women’s access to reproductive health services: a population-level study of Simiyu Region Tanzania. BMC Public Health.

[55] Sialubanje C, Massar K, van der Pijl MSG, Kirch EM, Hamer DH, Ruiter RAC. Improving access to skilled facility-based delivery services: Women’s beliefs on facilitators and barriers to the utilisation of maternity waiting homes in rural Zambia. Reprod Health [Internet]. 2015 Jul 8 [cited 2020 Sep 9];12. Available from: https://www.ncbi.nlm.nih.gov/pmc/articles/PMC4493824/. 10.1186/s12978-015-0051-6 PMC4493824 26148481

[56] Adjiwanou V, LeGrand T (2014). Gender inequality and the use of maternal healthcare services in rural sub-saharan Africa. Health Place.

[57] Mamun MAA, Hoque MM (2022). The impact of paid employment on women’s empowerment: a case study of female garment workers in Bangladesh. World Dev Sustain.

[58] Amwonya D, Kigosa N, Kizza J (2022). Female education and maternal health care utilization: evidence from Uganda. Reprod Health.

[59] Weitzman A (2017). The effects of women’s education on maternal health: evidence from Peru. Soc Sci Med 1982.

[60] O’Neil A, Russell JD, Thompson K, Martinson ML, Peters SAE (2020). The impact of socioeconomic position (SEP) on women’s health over the lifetime. Maturitas.

[61] Garrison-Desany HM, Wilson E, Munos M, Sawadogo-Lewis T, Maïga A, Ako O (2021). The role of gender power relations on women’s health outcomes: evidence from a maternal health coverage survey in Simiyu region, Tanzania. BMC Public Health.

