# Supplementary material for: Gender inequities in women’s access to maternal health care utilisation in Zambia: a qualitative analysis
Source: BMC Pregnancy Childbirth. 2023 Oct 26;23:755. doi: 10.1186/s12884-023-06078-3 (PMC10601225; doi:10.1186/s12884-023-06078-3)
Supplement: Supplementary file 1 — Additional file 1: Supplementary Materials 1, 2 and 3. [file 12884_2023_6078_MOESM1_ESM.docx]

**Supplementary materials**

**Supplementary material 1: Data Collection management**

The RAs were recruited based on their qualitative research skills and past experience. They needed to demonstrate basic skills in computers and use of audio recorders and were conversant with the primary languages spoken by the community to which they were assigned (Tonga and/or Bemba). Training of RAs was done in an entire day via zoom on ethics of data collection and COVID-19 safe data collection guidelines in accordance with the WHO and Zambia Ministry of Health guidelines. Sanitisation applied on all the data collection instruments such as paper, voice recorders, etc, wearing of masks and the social distance 1.5 meter rule applied during collection of data. The training also included orientation to the study in order to familiarise the team to the study aims and objectives. The RAs were guided step by step on how to recruit participants to the study and they also practiced in interviewing skills of the IDIs, recording and storage of interview recordings during training. These were reiterated throughout data collection to ensure research ethics were followed. The RAs also did role plays for the interviewers and interviewees during training to ensure that the research materials were properly understood.

In-depth participant interviews were conducted with women who gave birth in the last three years from both the health facility and at home as well as men whose wives/partners gave birth within the last three years from health facilities and at home to investigate maternal health care perceptions and personal experiences and to identify opportunities for improving maternal health services. Interview sessions lasted between 45 minutes and 1 hour and they were conducted at a place convenient for both the interviewer and interviewee within the community. Follow up interviews by the principal investigator were done to help validate perceptions and experiences of maternal health service utilisation.

The principal investigator ensured that there was quality of the data by conducting regular reflective discussions with the RAs. These discussions were held at the end of the day to discuss key findings, refine IDI guides, and identified strategies that continually enhanced the line of inquiry following the tradition of emergent design in qualitative research as well as identify what went well or not during data collection. Study participants were probed to elaborate on their responses to make sure their views were correctly captured. Throughout data collection and transcription, we ensured confidentiality of the data by ensuring that individual personal identifiers were not collected and authors did not have access to information that could identify individual participants during or after data collection. The RAs who spoke the local languages translated the interview guide in local languages (Bemba and Tonga) for the participants and then back-translated the answers into English.

**Supplementary material 2: Participants demographic factors by age and district**

| **District** | **Age group** | **Women** | **Men** |
| --- | --- | --- | --- |
| Kalomo | 15-19 | 2 | 0 |
| Kalomo | 20-24 | 3 | 1 |
| Kalomo | 25-29 | 2 | 1 |
| Kalomo | 30-34 | 2 | 0 |
| Kalomo | 35-39 | 1 | 1 |
| Kalomo | 40-44 | 1 | 2 |
| Kalomo | 45-49 | 1 | 1 |
| Mansa | 15-19 | 1 | 0 |
| Mansa | 20-24 | 3 | 0 |
| Mansa | 25-29 | 3 | 0 |
| Mansa | 30-34 | 3 | 2 |
| Mansa | 35-39 | 2 | 2 |
| Mansa | 40-44 | 0 | 1 |
| Mansa | 45-49 | 0 | 1 |
| **Total** |  | **24** | **12** |

**Supplementary material 3: Gender analysis framework: Understanding gender as a power relation and driver of inequality in maternal health care utilisation**

| **What constitutes gendered power relations?** | Who has what? | Access to resources (education, information, skills, income, employment, services, benefits, time, space, social capital, distance to health facilities)   - Indirect costs associated with accessing antenatal care, delivery (birth supplies and baby needs) and postnatal - Transportation to the health facility and for referrals - Nutrition during pregnancy and breastfeeding - Sufficient health care staff - Readily available and appropriate hospital equipment - Health facility structure and equipment - Adequate, appropriate and quality health care - Literacy to understand maternal health care packages |
| --- | --- | --- |
|  | Who does what? | Division of labour within and beyond the household and every day practices   - Maternal health care utilisation - Household chores - Who brings family income |
|  | How are values defined? | Social norms, ideologies, beliefs and perceptions   - Decision to seek maternal health care - Choice of birth place - Perception of quality of health care |
|  | Who decides? | Rules and decision-making (formal and informal)   - Who makes decisions on whether or not to seek health care - Who makes decision on where to deliver from, husband/family/resources |
| **How is power negotiated and changed?** | Individuals/people | Critical consciousness, acknowledgement (or lack of), agency/apathy, interests, historical and lived experiences, resistance, violence   - Relationship between husband and wife - Past maternal health experience - Relationship between wife, husband and health facility staff |
|  | Structural/environmental | Legal and policy status, institutionalism within planning and programs, funding, accountability mechanisms   - How free is maternal health care (indirect costs despite it being free by national policy) - Presence of husband/family during antenatal, delivery and postnatal care |
